# Multidisciplinary Management of Single Organism Emphysematous Splenitis Without Splenectomy: A Case Report

**DOI:** 10.7759/cureus.80389

**Published:** 2025-03-11

**Authors:** Suanne C MacConnell, Anand Trivedi

**Affiliations:** 1 General Surgery, Fiona Stanley Hospital, Perth, AUS

**Keywords:** abscess, antimicrobial therapy, clostridium perfringens, percutaneous drainage, spleen

## Abstract

Emphysematous splenitis traditionally requires a splenectomy, resulting in life-long consequences for the patient. *Clostridium perfringens* is often seen in an immunocompromised population. This case demonstrates a multidisciplinary team approach consisting of percutaneous drainage and a prolonged intravenous and oral antibiotic regime, including ceftriaxone and metronidazole, as well as amoxicillin and clavulanic acid, to provide a successful outcome in an elderly immunocompetent female. This negated the need to proceed to the traditional operative management of splenectomy and validates an equitable conservative approach to treat a *C. perfringens*-induced emphysematous splenic infection. This approach was undertaken and was likely successful secondary to the haemodynamic stability and immunocompetent baseline of the patient. Her immunocompetency could be maintained due to the non-operative management and should therefore be considered if the clinical situation allows.

## Introduction

Splenic abscesses have an incidence of 0.14-0.7% throughout autopsy reports worldwide [1,2]. Emphysematous splenitis is an even rarer condition, with predisposing factors for splenic infection described as trauma, malignancy, immunosuppression, and systemic infection with haematological spread [2,3]. Causative agents have been reported to include *Escherichia coli, Proteus mirabilis,* *Klebsiella pneumonia, Pseudomonas *spp, and *Staphyloccous aureus* [1,3,4]. *Clostridium perfringens* as the primary cause is an even rarer entity, with only eight previously published cases [2]. Diagnosis is challenging secondary to the non-specific symptoms [1] but can include left upper quadrant abdominal pain, fever, tachycardia, vomiting, diarrhoea, and splenomegaly [1,5-8]. The poorly specific signs and symptoms and the uncommon nature of splenic infections necessitate cross-sectional imaging for diagnosis [5,9]. Computer tomographic (CT) imaging of the abdomen can be utilised to define differential diagnoses, including viral, fungal, or bacterial infection, haematoma, lymphoproliferative disorders, or metastases [9]. CT imaging is also vital in defining the complications of splenic infections, including infarction, haemorrhage, rupture, or splenic artery aneurysm. Despite the importance of imaging, clinical history and laboratory findings must not be overlooked [9] as changes to the clinical picture often drive management decisions. Traditionally, emphysematous splenitis has been managed with a splenectomy [1-5], but recent evidence has proven the non-operative approach successful [10-12]. The operative approach results in an asplenic patient producing a multitude of life-long concerns pertaining to infection risk [13]; hence, if the case and evidence allow, non-operative alternatives should be explored. The evidence for non-operative management however remains slight, and therefore a multidisciplinary patient-centred approach is suggested.

## Case presentation

History of presenting complaint

An independent 83-year-old female presented with a history of increasing left abdominal/flank pain and vomiting over a few days. She denied fevers but reported extreme fatigue, weakness, dizziness, and an increasing shortness of breath. She denied being unwell recently; specifically, she did not report night sweats, weight loss, or overseas travel. Her background consisted of traumatic left-sided rib fractures, which required no intervention over five years ago, hypertension, dyslipidemia, and an open appendicectomy. On review by the surgical team, the patient was found to be comfortable, with observations of a heart rate of 93 bpm, blood pressure of 123/105 mmHg, and afebrile. Her abdomen was soft, with tenderness to her left upper quadrant but no guarding.

Investigations

**Figure 1:**
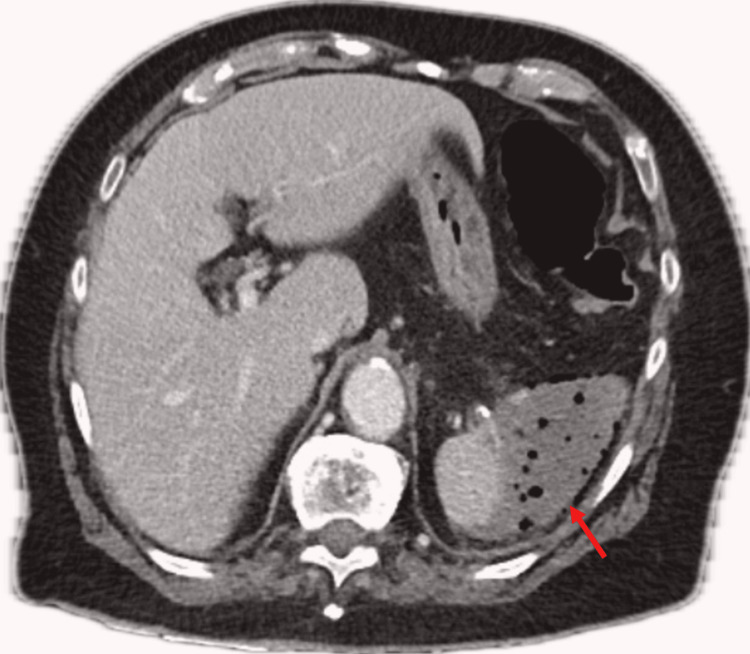
Emphysematous spleen on CT imaging at presentation (red arrow)

Her biochemical markers on arrival demonstrated a white cell count (WCC) of 30.4 x 10^9^/L (Table 1). Her C-reactive protein (CRP) was found to be raised at 218 mg/L, and she had an acute kidney injury with a creatine of 114 micromol/L (Table 1). Her lactate was elevated at 3.3 mmol/L, thought secondary to sepsis (Table 1). Her HbA1c was found to be normal at 5.9% (Table 1). No evidence of an immunocompromised state was able to be established. Cross-sectional imaging with computed tomography (CT) revealed an emphysematous splenic collection involving most of the splenic parenchyma (Figure 1) and non-enhancing splenic vein branches at the hilum of uncertain aetiology.

**Table 1:** Laboratory results throughout the management course

	Patient (on admission)	Patient (on discharge)	Patient (follow-up)	Normal range
White cell count (WCC) (x10^9^/L)	30.4	8.78	6.28	4.5-11
C-reactive protein (CRP) (mg/L)	218	71	3.6	<3
Creatinine (micromol/L)	114	40		45-90
Lactate (mmol/L)	3.3			<1
HbA1c (%)	5.9			<5.7

Treatment and outcome

Surgical

**Figure 2:**
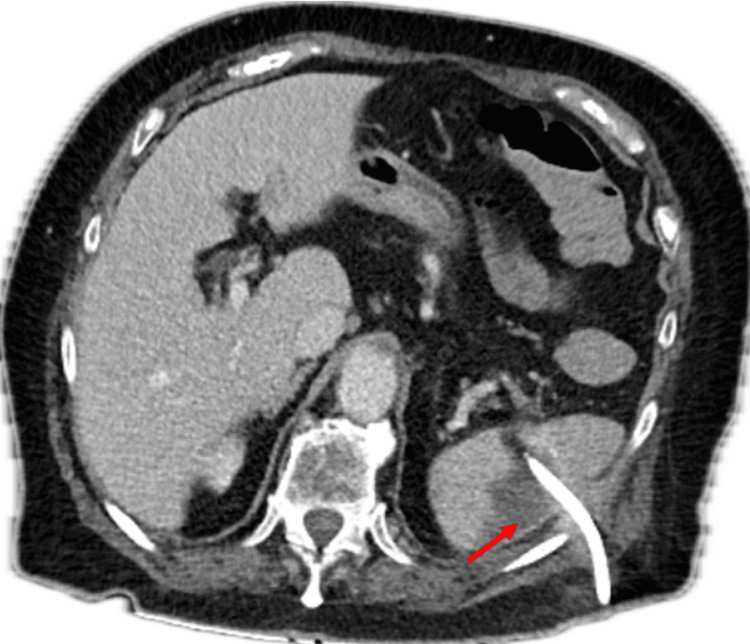
CT post-interventional imaging-guided drainage demonstrating a reduction in collection prior to discharge (red arrow)

A decision was made to offer percutaneous drainage of the splenic abscess given the patients’ haemodynamic stability and unusual presentation, which was performed by the interventional radiologists. Microscopy, culture, and sensitivity (MC&S) samples grew abundant pure growth of *C. perfringens,* which was sensitive to penicillin, metronidazole, and clindamycin. Blood cultures throughout the stay remained negative. The patient required a 12-day admission with involvement from the infectious disease department (IDD) to steer antimicrobial therapy with initial treatment consisting of intravenous (IV) antibiotics and the use of clindamycin reserved for any clinical deterioration. Recommendations were made by the infectious disease team to proceed with a splenectomy; however, the surgical team did not deem this necessary given the patient’s stable clinical condition and reduction of her WCC to 11.8 x 10^9^/L and CRP to 115 mg/L by day five of admission. Imaging was performed prior to discharge, demonstrating resolution of the emphysematous component and reduction in the collection (Figure 2).

Antimicrobial

A peripherally inserted central catheter was placed, and IV ceftriaxone 2 g daily and metronidazole 500 mg twice daily were continued for six weeks. A further six weeks of oral antibiotics consists of amoxicillin 875 mg and clavulanic acid 125 mg twice daily with an additional 1 g of amoxicillin daily. This prolonged course of antibiotics was felt necessary given the non-operative approach. No adverse side effects were experienced by the patient with notable reduction of her high inflammatory markers (Table 1) and progressive resolution of the splenic abscess.

Follow-up

**Figure 3:**
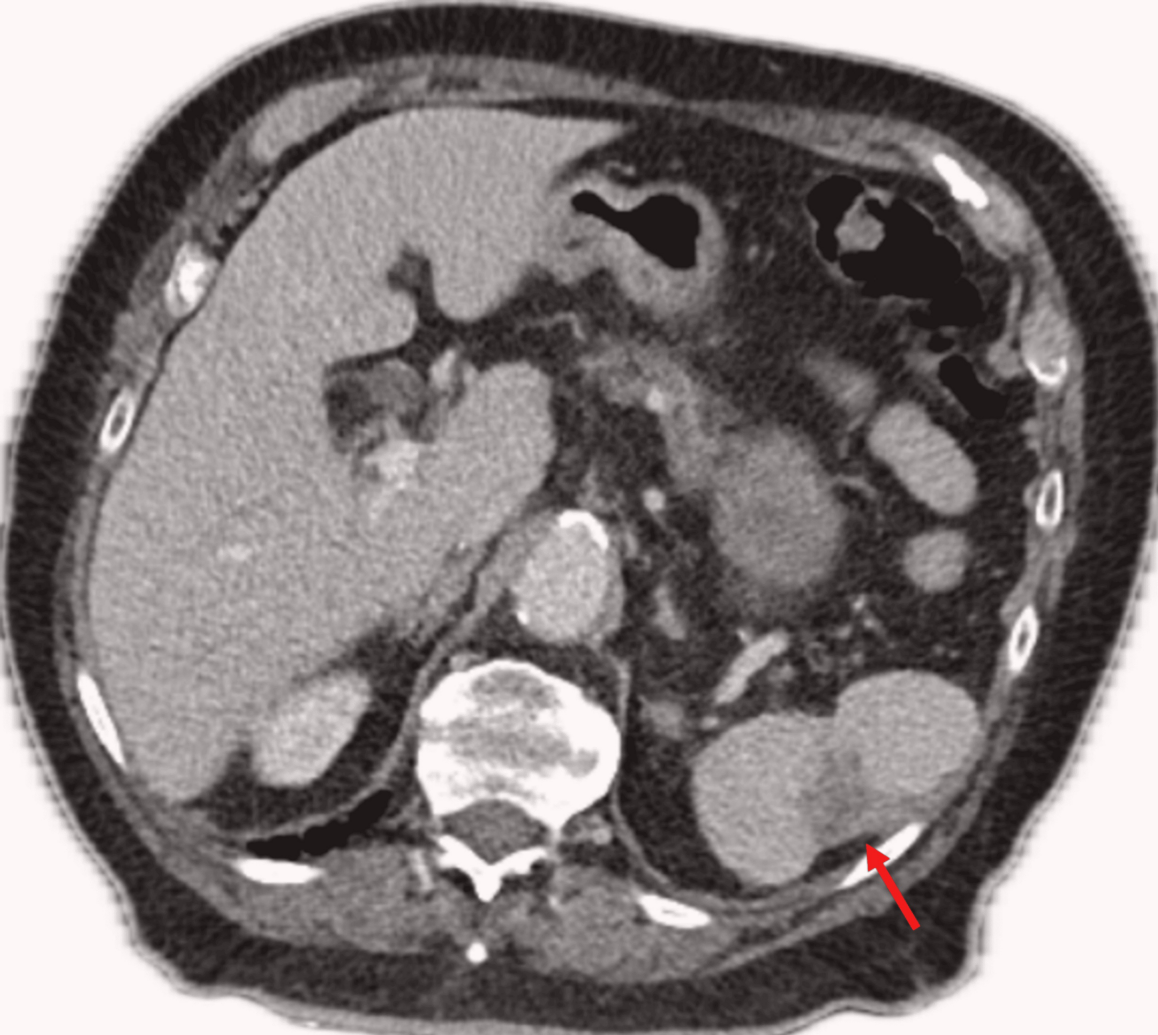
Stable CT appearance at two-month post presentation (red arrow)

Surgical follow-up consisted of outpatient appointments with a CT at the two-month mark revealing stable appearance of the spleen and no undrained collection (Figure 3). The pigtail catheter was removed, and the patient was discharged. The patient was referred for a colonoscopy in attempt to identify the source of *C. perfringens*; however, this was normal.

Follow-up from the IDD consisted of regular clinic appointments to assess the patient’s symptoms and biochemical markers (Table 1) and to monitor the extended course of antimicrobial therapy. The patient was last reviewed eight months post initial presentation where she reported to be well with no evidence of persistent or recrudescent infection despite being off antibiotics for the previous four months. There was no evidence of hyposplenism on the blood film, and the patient was subsequently discharged.

## Discussion

Pathogenesis

Splenic abscesses have an incidence of 0.14-0.7% and are commonly identified in patients who have sustained trauma, malignancy, or immunodeficiency [1-3]. *C. perfringens* is commonly present in immunocompromised patients [4] and is a rare cause of splenic abscesses with only eight other published cases [2], with our case appearing to be the only successful non-operative management. Given our patient had no risk factors, the working diagnosis from all involved was that of an infarcted spleen, although it is difficult to ascertain whether this is a cause or consequence of the infection. Clostridium myonecrosis has been demonstrated to alter blood flow to the affected tissue [14] and has been proven to cause deadly infection in those without previous malignancy or traumatic events [15].

Management decision

Previous reports of gangrenous spleen necrosis have resulted in splenectomy [1-5], and there were members of the team who deemed this necessary to obtain source control. Non-operative approaches have been described previously for splenic abscesses and septic shock [2,4,11,12]. However, to the best of our knowledge, only one other published study has demonstrated successful management without splenectomy of an emphysematous infection [10]. This patient, in contrast to our case, was immunocompromised with diabetes and had *E. coli* cited as the source [9]. The antibiotic regime was a novel concept, but previous reports recorded successful treatment with IV metronidazole [2] and ceftriaxone [3] and subsequent oral step-down of amoxicillin and clavulanic acid [5]. This was therefore deemed feasible and applied in this case secondary to the haemodynamic stability and immunocompetent baseline of the patient. This ensured a reduction in susceptibility to severe and invasive infections and avoided the immunocompromising morbidity of a splenectomy [13].

## Conclusions

Emphysematous splenic infections may be managed conservatively without proceeding to an operative splenectomy. Treatment through a multi-disciplinary team approach consisting of percutaneous drainage and prolonged anti-microbial therapy while being overseen by the general surgical team proved successful in this case. Close observation, both biochemical and radiological, is recommended with the removal of the drain at an appropriate time frame. Conservative management should therefore be considered in appropriate cases to reduce the life-long burden of a splenectomy.
